# Temporal regulation of interferon signalling in human EndoC-βH1 cells

**DOI:** 10.1530/JME-21-0224

**Published:** 2022-04-19

**Authors:** Shalinee Dhayal, Kaiyven Afi Leslie, Mohammad Baity, Pouria Akhbari, Sarah J Richardson, Mark A Russell, Noel G Morgan

**Affiliations:** 1 Islet Biology Group (IBEx), Exeter Centre of Excellence in Diabetes (EXCEED), Institute of Biomedical & Clinical Science, University of Exeter Medical School, Exeter, UK

**Keywords:** Beta-cell, insulitis, STAT, type 1 diabetes, islets of Langerhans

## Abstract

During the development of type 1 diabetes, interferons (IFN) are elaborated from islet-infiltrating immune cells and/or from virally infected β-cells. They act via specific receptors to increase, acutely, the phosphorylation of the transcription factors STAT1 and 2. However, the longer-term impacts of chronic IFN stimulation are poorly understood and were investigated in the current study. Human EndoC-βH1 cells were treated with IFNα, IFNγ or IFNλ either acutely (<2 h) or chronically (≥24 h) and STAT phosphorylation, expression and activity were assessed by Western blotting and transcriptional reporter assays. Exposure of β-cells to IFNα or IFNλ induced a swift increase in the phosphorylation of both STAT1 and STAT2, whereas IFNγ increased only pSTAT1. Over more extended periods (≥24 h), STAT phosphorylation declined but STAT1 and STAT2 expression were enhanced in a sustained manner. All IFNs stimulated ISRE transcriptional activity (but with different time courses), whereas GAS activity was responsive only to IFNγ. The re-addition of a second bolus of IFNα, 24 h after an initial dose, failed to cause renewed STAT1/2 phosphorylation. By contrast, when IFNγ was added 24 h after exposure to IFNα, rapid STAT1 phosphorylation was re-initiated. Exposure of β-cells to IFNs leads to rapid, transient, STAT phosphorylation and to slower and more sustained increases in total STAT1/2 levels. The initial phosphorylation response is accompanied by marked desensitisation to the cognate agonist. Together, the results reveal that the response of β-cells to IFNs is regulated both temporally and quantitatively to achieve effective signal integration.

## Introduction

The progression of type 1 diabetes is associated with the development of inflammation in the islets of Langerhans, leading to the local production of cytokines which influence the function and viability of β-cells (Eizirik *et al.* 2009, Kaddis *et al.* 2015, Pugliese 2016, Donath *et al.* 2019, Demine *et al.* 2020, Leete & Morgan 2021). These cytokines derive both from the influx of immune cells targeting specific islet autoantigens and from the β-cells themselves, which establish an active molecular dialogue with the immune system and with neighbouring islet cells (Ventriglia *et al.* 2015, Craig *et al.* 2019, Bender *et al.* 2020, Erdem *et al.* 2021). Among the cytokines present in the islet milieu in type 1 diabetes are a variety of interferons (IFN) including the type I interferon, IFNα, which is likely to emanate primarily from the β-cells themselves (Foulis *et al.* 1987). IFNα is induced in response to viral infection and there is now substantial evidence implicating the establishment of a persistent enteroviral infection in the β-cells during an early phase of the disease process (Alidjinou *et al.* 2014, Jean-Baptiste *et al.* 2017, Qaisar *et al.* 2018, Dunne *et al.* 2019, Akhbari *et al.* 2020, Marroqui *et al.* 2021). In addition, recent data imply that type III IFNs (IFNλ1 and λ2) are also likely to be present in the vicinity of islet cells, as is IFNγ, produced by influent immune cells (Lind *et al.* 2013, Colli *et al.* 2018, 2020). Thus, as type 1 diabetes progresses, pancreatic β-cells are exposed to a variety of IFNs and, to marshal an effective response, the cells must interpret and integrate these input signals effectively.

The signal transduction mechanisms elicited by each class of IFNs have been studied in detail in many cell types and their responses are increasingly well-characterised in β-cells (Wesoly *et al.* 2007, Gough *et al.* 2008, Lopez de Padilla & Niewold 2016, Yan *et al.* 2018). Accordingly, it is understood that their actions share certain characteristic features including the activation of specific cell surface receptors which leads, in turn, to the recruitment and activation of associated Janus Kinases. These then phosphorylate the receptors on specific tyrosine residues leading to the recruitment and subsequent phosphorylation of defined subsets of transcription factor belonging to the signal transducer and activator of transcription (STAT) family. In the case of type I and type III IFNs, STAT1 and STAT2 are recruited to the receptors and phosphorylated by Jak1 and Tyk2 whereas, for IFNγ, STAT1 is the primary target. Upon phosphorylation, the STAT molecules dimerise to form either homo- (pSTAT1) or hetero-dimers (pSTAT1/pSTAT2) which then further recruit additional binding partners before translocation to the nucleus and activation of transcription (Majoros *et al.* 2017, Mazewski *et al.* 2020).

Despite this clear delineation of the early events involved in mediating IFN responses in β-cells, it is much less clear how the sustained actions of IFNs are regulated and the extent to which these different signalling pathways are integrated to generate cellular responses during exposure to multiple IFNs. It is important, therefore, to develop a more complete understanding of these processes to allow effective targeting of IFN-responsive pathways and to facilitate the development of an immunotherapeutic armoury designed to attenuate β-cell loss in type 1 diabetes. In the present study, we have addressed this gap in understanding and have examined the temporal changes associated with IFN action in human β-cells using the EndoC-βH1 cell as a model (Ravassard *et al.* 2011). In addition, we have also studied the interactions between the various IFN subtypes to gain more complete picture of the integrated cellular responses.

## Materials and methods

### Materials

DMEM (25 mmol/L glucose), DMEM (5.5 mmol/L glucose), NuPAGE® Novex® Bis-Tris Gels, NuPAGE® LDS sample buffer (4×) and anti-STAT2 were acquired from Thermofisher Scientific. BSA, fibronectin, transferrin, sodium selenite, nicotinamide, Tris, NaCl, EDTA, Triton-X100, protease inhibitor, phosphatase inhibitor cocktails 2 and 3, IGEPAL, PVDF membrane, paraformaldehyde and protein G sepharose beads were purchased from Merck. Gamma-interferon activation site (GAS) and interferon stimulation response element (ISRE) reporter construct were from Qiagen. Mirus TransIT-2020 transfection reagent was obtained from Cambridge Bioscience (Cambridge, UK). Anti-STAT1 was obtained from Cell Signalling Technology. Anti-phospho STAT1 and anti-phospho STAT2 were acquired from Abcam. Anti-GAPDH was from Proteintech (Manchester, UK). Isotype control anti-mouse IgG was from Dako.

### Cell culture

The Human pancreatic β-cell line EndoC-βH1 (Ravassard *et al.* 2011) was grown in a monolayer on Matrigel-fibronectin-coated plates (coating medium – DMEM 25 mmol/L glucose supplemented with 2 μg/mL fibronectin and 1% (v/v) extracellular matrix). Cells were cultured in DMEM containing 5.5 mmol/L glucose, 2% (w/v) BSA (fraction V), 50 μmol/L β-mercaptoethanol, 5.5 μg/mL transferrin, 6.7 ng/mL sodium selenite, 10 mM nicotinamide, penicillin (100 units/mL) and streptomycin (100 µg/mL) and maintained at 37°C, 100% humidity and 5% CO_2_ (Andersson *et al.* 2015).

### Western blotting and co-immunoprecipitation

EndoC-βH1 cells were grown at 2.5 × 10^5^/mL and after treatment with interferons, whole-cell protein was extracted in lysis buffer containing 20 mmol/L Tris (pH 8); 150 mmol/L NaCl; 1 mmol/L EDTA and 1% (v/v) Triton X-100. This was supplemented with 10 µg/mL protease inhibitor and 10 µg/mL phosphatase inhibitor cocktails 2 and 3 before use. Protein samples were denatured and run on 4–12% SDS PAGE gradient gels. Following protein transfer, PVDF membranes were probed with anti-phospho STAT1 (Abcam #ab29045; 1 in 1000 dilution); anti-total STAT1 (Cell Signalling #14994; 1 in 1000 dilution), anti-phospho STAT2 (Abcam #ab53149; 1 in 1000 dilution) or anti-total STAT2 (Thermofisher Scientific #44-362G; 1 in 1000 dilution). GAPDH (Proteintech #60004-1; 1 in 10,000 dilution) was examined as a loading control. To ensure that our extraction procedures were successful in harvesting both nuclear and cytosolic proteins, some samples were also probed with antiserum raised against histone H3 (not presented).

For immunoprecipitation, whole-cell protein was extracted from 10^6^ cells in lysis buffer containing 50 mmol/L Tris (pH 8); 150 mmol/L NaCl; 1 mmol/L EDTA and 1% (v/v) IGEPAL supplemented with protease and phosphatase inhibitors (as above) before use. The lysates were incubated with 3 µg of anti-total STAT1 (Cell signalling #9176) or isotype control mouse IgG (Dako; #X0931) overnight at 4°C. Protein G sepharose beads were then added for 4 h at 4°C followed by three washes with lysis buffer. Proteins were eluted using 1× LDS sample buffer and 10% (v/v) β-mercaptoethanol at 70°C for 10 min. Western blot analysis was then performed as described above.

### Immunocytochemistry

EndoC-βH1 cells were seeded at a density of 4 × 10^5^/mL and left to adhere for 48 h. Following appropriate treatment, cells were fixed with 4% paraformaldehyde. Fixed cells were permeabilised with buffers containing 0.2% Triton (0.1 M lysine, 10% donor calf serum, 0.02% sodium azide, in PBS) for 30 min prior to staining with either anti-total STAT1 (Cell Signalling #14994; 1 in 200 dilution) and/or anti-total STAT2 antibody (Thermofisher Scientific #44-362G; 1 in 200 dilution). Images were captured using a Leica DM4000 B LED Fluorescence microscope.

### Dual luciferase assay

EndoC-βH1 cells were plated at 2 × 10^5^ cells/mL and left to adhere for 4 h, before transfection with ISRE or GAS luciferase reporter constructs (400 ng/mL) using TransIT-2020 transfection reagent (4 µL/1 µg DNA). Cells were incubated for 4 h and then treated with the appropriate interferons and incubated for a further 24 h. At the end of the incubation period, cells were lysed for 45 min at room temperature. Luciferase activity was measured using a PHERAstar microplate reader (BMG LABTECH). Luciferase activity was normalised to the vehicle-treated control (defined as 1.0) and expressed as relative change (fold-change) from the control value. Statistical analysis was performed on the transformed values.

### Statistical analysis

Data are expressed as mean values ±s.e.m. Where a pair of experimental groups were compared, statistical significance was calculated using Student’s *t*-test. Alternatively, when more than two groups were examined, one-way ANOVA was employed with Bartlett* post hoc* test. *P*  < 0.05 was considered statistically significant.

## Results

### Temporal regulation of transcriptional events in beta-cells following interferon treatment

**Figure 1:**
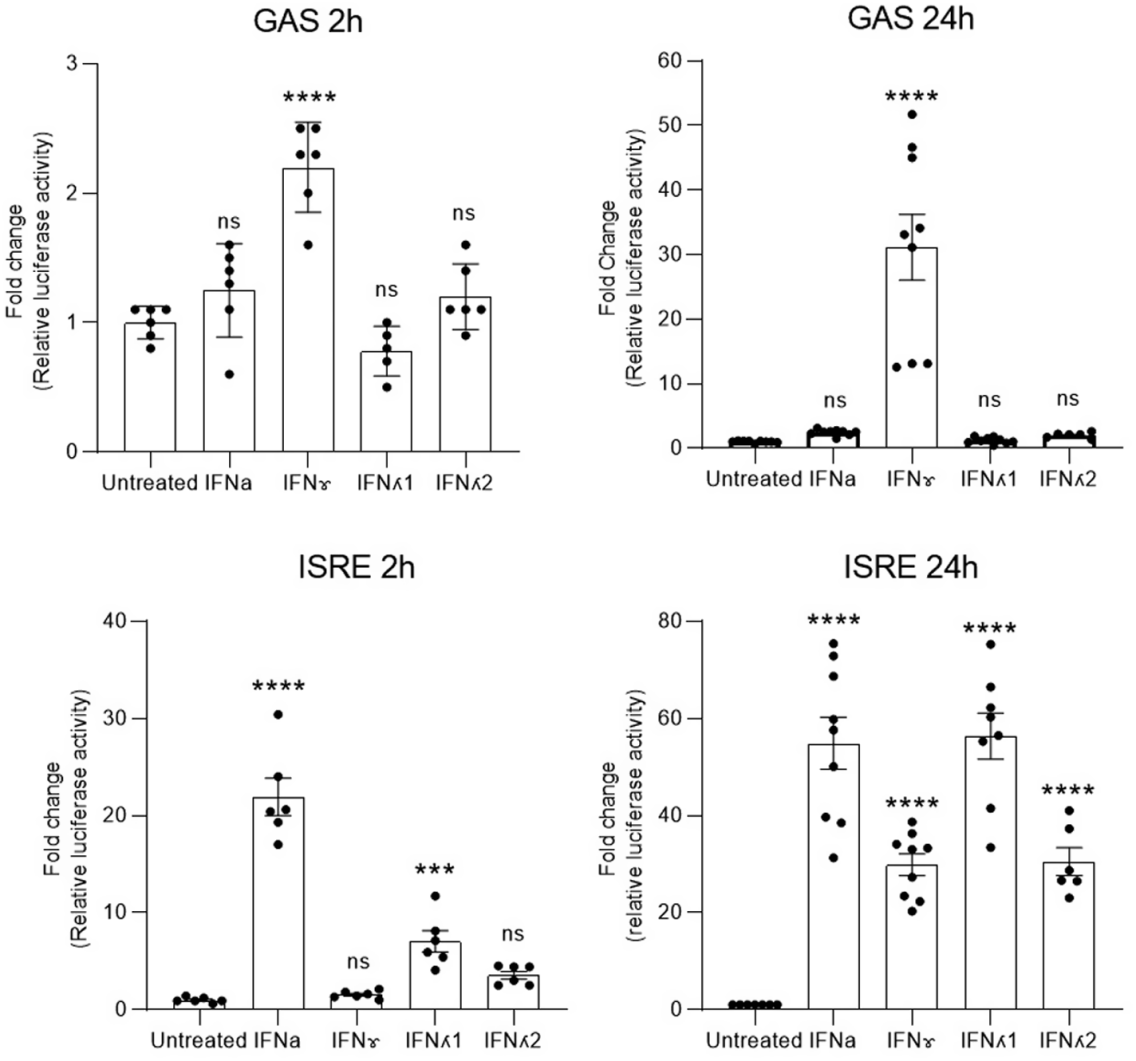
EndoC-βH1 were seeded at a density of 2 × 10^5^/mL and incubated for 4 h, after which the cells were transfected with ISRE or GAS reporter and incubated for a further 4 h. The cells were then treated with appropriate interferons and incubated for 24 h prior to measurement of luciferase activity. ^****^P  < 0.0001; ^***^P  < 0.001; ns, not significant.

Initial studies focussed on the time-dependence of transcriptional events initiated upon the addition of type I, II and III interferons to EndoC-βH1 cells. For these experiments, cells were transfected with luciferase reporter constructs under the control of either a GAS or ISRE promoter and the luciferase activity was then measured after a further 2 or 24 h following the addition of interferon (Fig. 1). The luciferase activity of the GAS reporter construct was increased significantly within 2 h of addition of IFNγ but was unaffected by exposure of the cells to either type I or type III IFNs. A similar selectivity was maintained during more extended exposure (up to 24 h) although the magnitude of the response to IFNγ had increased dramatically (from ~2- to >30-fold) at this later time. By contrast, the responses mediated by the ISRE reporter were less selective and showed a more variable time course. Thus, although a modest increase in activity was induced by IFNλ1 within 2 h (reaching ~5-fold above basal), this was further increased (to reach 55-fold) at 24 h. IFNα also caused a more than 50-fold activation of the ISRE reporter at 24 h but this agent was much more efficacious than IFNλ1 at the 2 h time point; by which it had already provoked a 20-fold rise in reporter activity.

Addition of IFNλ2 caused a small, but not statistically significant, rise in ISRE-driven reporter activity within 2 h, whereas this had increased to ~30-fold by 24 h. Unexpectedly, IFNγ-induced ISRE-driven reporter activity had also increased by almost 30-fold at the 24 h time point, despite the fact that this agent did not elicit any ISRE-driven response within 2 h. Thus, while the responses mediated by a GAS-driven construct were regulated uniformly in EndoC-βH1 cells, there were both temporal and quantitative differences in the responses caused by all IFNs in cells transfected with a construct encoding an ISRE-promoter.

### Temporal regulation of STAT phosphorylation and expression in beta-cells following interferon treatment

**Figure 2:**
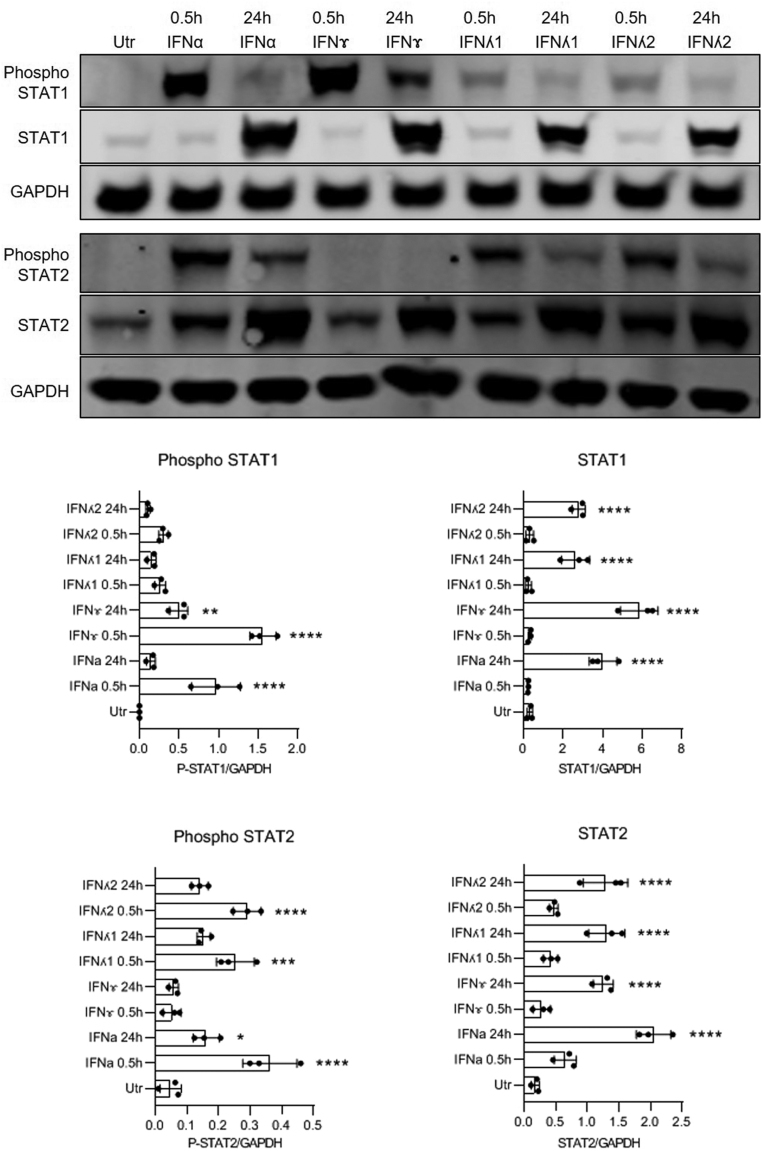
EndoC-βH1 cells were treated with various interferons (IFNα – 1000 U/mL; IFNɤ – 20 ng/mL; IFNʎ1 or 2 – 200 ng/mL) for either 30 min or 24 h. Protein was extracted at the end of the incubation and Western blotting performed using anti-phospho STAT1, anti STAT1, anti-phospho STAT2 and anti STAT2. A representative blot is shown for each target protein, but densitometric traces were obtained from a minimum of three separate experiments in each case (lower panels). GAPDH was used as a loading control. ^****^P  < 0.0001; ^**^P  < 0.01; ^*^P  < 0.05.

In an attempt to understand these differences more completely, we next monitored both the tyrosine phosphorylation of relevant STAT isoforms (STAT1 and STAT2) and the extent of protein expression at early and later time points in EndoC-βH1 cells exposed to each of the four IFNs (Fig. 2). This again revealed important variations. For example, IFNγ caused a rapid (within 30 min) and profound increase in STAT1 phosphorylation which had declined by ~70% at 24 h but was still elevated above control at this time. Moreover, as anticipated, STAT2 phosphorylation was not increased in cells exposed to IFNγ at either time point studied. Similarly, IFNα also caused an early and marked increase in STAT1 phosphorylation in EndoC-βH1 cells but this had declined by ~90% at 24 h. However, unlike IFNγ, IFNα also promoted the phosphorylation of STAT2 within 30 min and the response was more sustained than the phosphorylation of STAT1, since STAT2 phosphorylation was still elevated significantly at 24 h. IFNλ1 also caused an early and large increase in STAT2 phosphorylation in EndoC-βH1 cells and this was only marginally smaller in magnitude than that provoked by IFNα. The response to IFNλ1 then declined but was still evident at 24 h. IFNλ1 caused only a small and transient increase in STAT1 phosphorylation and an essentially identical profile of responses was seen when cells were exposed to IFNλ2 (Fig. 2). The magnitude of these effects was reduced compared to that seen with IFNα but the early STAT1 phosphorylation provoked by both IFNλs could be seen more readily with longer exposure of the blots (not presented).

**Figure 3:**
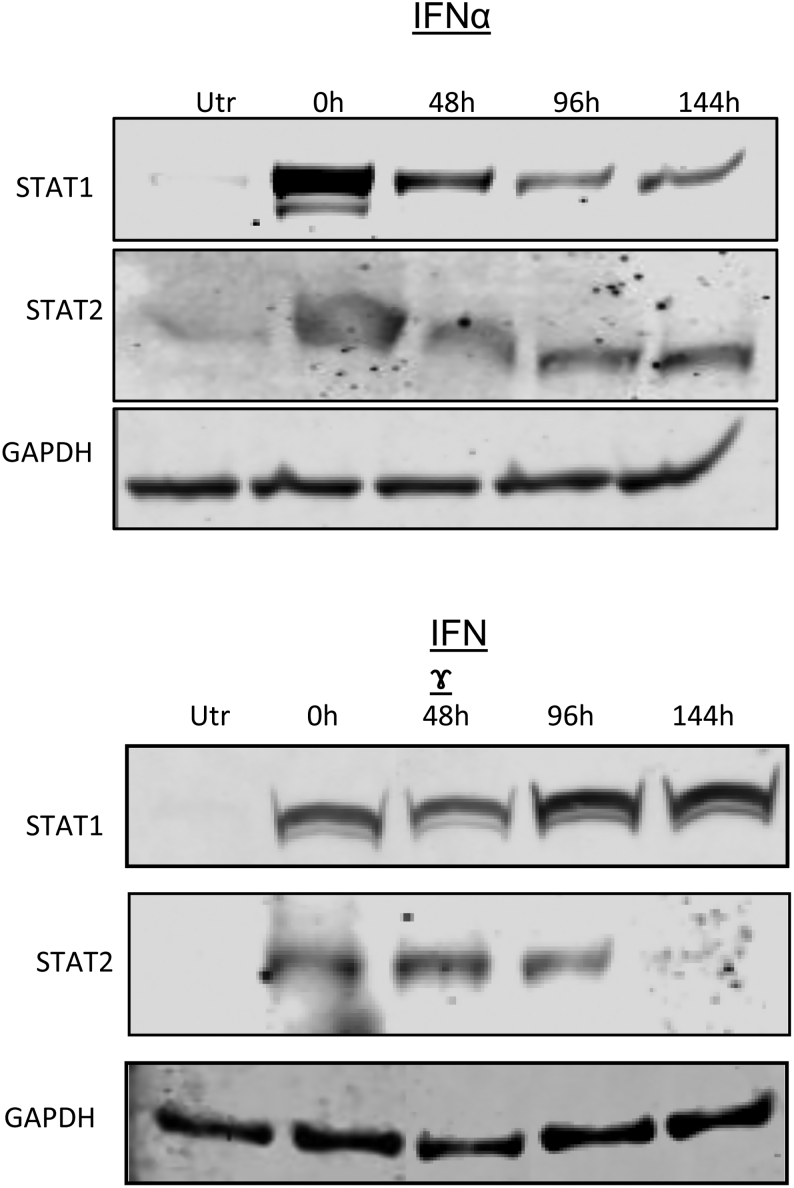
EndoC-βH1 were treated with IFNα (1000 U/mL) or IFNɤ for 24 h, as shown. The cells were washed with PBS and the medium replaced with normal medium (lacking interferons) and cultured for increasing periods of time (24, 48, 96 and 144 h). Protein was extracted at each time point and Western blotting performed using anti-STAT1 and anti-STAT2 sera as shown. Representative blots are presented and GAPDH was used as a loading control.

In parallel with the alterations in STAT isoform phosphorylation, we also monitored the total expression of both STAT1 and STAT2 in cells exposed to each IFN since this was, again, found to vary (Fig. 2). Most significantly, it was noted that the expression of both STAT1 and STAT2 became upregulated dramatically within 24 h when EndoC-βH1 cells were exposed to each of the IFN subtypes. This effect was, at least in part, independent of the ability of each IFN to promote the tyrosine phosphorylation of its cognate STAT isoforms, since STAT2 levels were markedly increased in cells treated with IFNγ, even though this agent did not induce the phosphorylation of STAT2. We also found that the induction of total STAT1 was sustained long after phosphorylation had declined to baseline during exposure to either IFNα or IFNγ. For these experiments, cells were exposed initially to IFNα or IFNγ for 24 h and then washed to remove the stimulus (Fig. 3). They were then subsequently incubated in fresh medium (with no added IFNs) for various additional periods prior to harvesting and lysis. Control cells which had not been exposed to IFNs retained minimal levels of STAT1 and STAT2 during these studies. However, those cells which had been treated for 24 h with either IFNα or IFNγ retained an elevated level of STAT1 and STAT2 for at least a further 6 days. This elevation was sustained most effectively in cells initially exposed to IFNγ (Fig. 3). Overall, these studies revealed a complex set of interrelationships between changes in STAT isoform phosphorylation and STAT expression during exposure of EndoC-βH1 cells to IFNs.

### Interactions between STAT isoforms in beta-cells following interferon treatment

**Figure 4:**
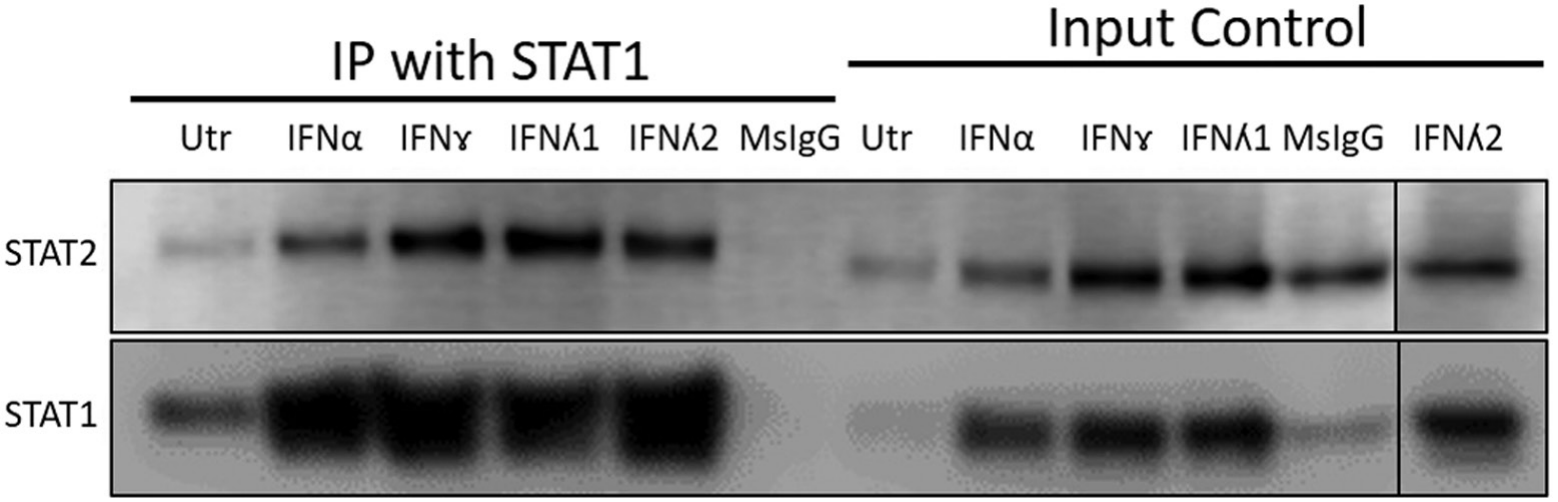
EndoC-βH1 were treated with IFNα (1000 U/mL) or IFNγ (20 ng/mL) or IFNʎ1 or 2 (200 ng/mL) for 24 h. After this period, cells were lysed and 2% of the input lysate removed, denatured and stored at −20°C. The remainder of the protein lysate was incubated with 2 µg of STAT1 antibody or 2 µg of isotype control IgG overnight at 4°C. A 50% bead slurry was added, proteins eluted and Western blotting was performed using antisera directed against either STAT2 and STAT1.

**Figure 5:**
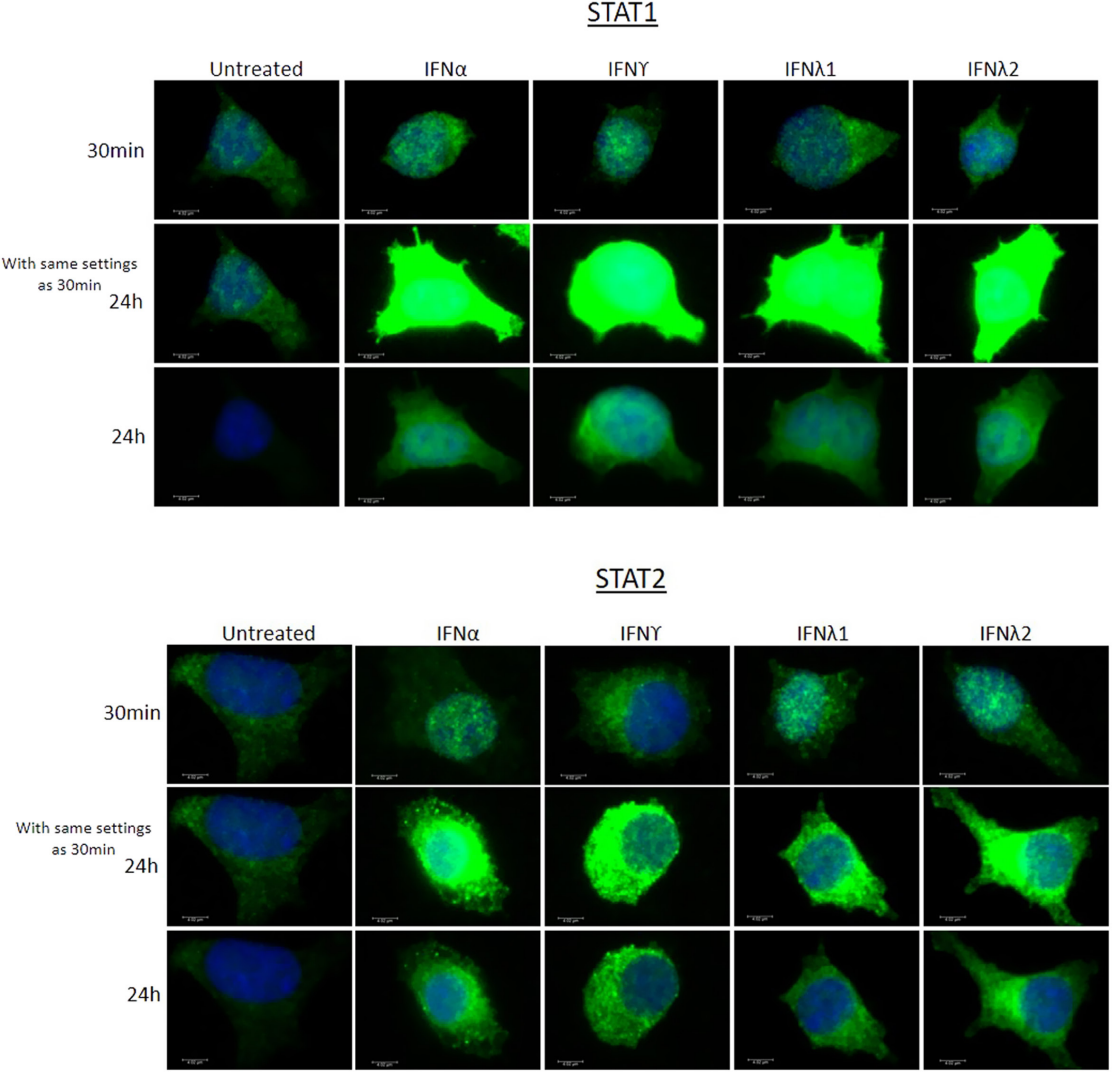
EndoC-βH1 treated with either 1000 U/mL IFNα, 20 ng/mL IFNγ or 200 ng/mL IFNʎ1 or IFNʎ2 for 30 min. Cells were fixed with 4% paraformaldehyde, permeabilised and stained with anti-STAT1 or anti-STAT2 antisera, as shown. Images were taken using a Leica DM4000 B LED Fluorescence microscope using exposure settings defined for the control conditions (upper and middle panels of each figure). In the lower panels of each figure, the exposure settings were adjusted to provide improved resolution of the subcellular localisation of each STAT isoform after incubation with interferons.

Intrigued by the finding that IFNγ caused a marked increase in ISRE-driven reporter activity despite its failure to promote STAT2 phosphorylation, we then employed an immunoprecipitation approach to investigate whether IFNγ might induce complex formation between STAT1 and STAT2 in EndoC-βH1 cells (Fig. 4). Thus, cells were exposed to IFNs and 24 h later, they were lysed and an antibody directed against STAT1 used to immunoprecipitate any cognate protein complexes formed in the cells under these conditions. As anticipated, treatment of cells with type I and type III IFNs resulted in the formation of a complex containing both STAT1 and STAT2 since the latter was detected in the immunoprecipitate pulled down with anti-STAT1 in all cases. Moreover, to our surprise, STAT2 was also pulled down in parallel with STAT1 when cells were exposed to IFNγ. Thus, despite the failure to detect STAT2 phosphorylation in response to IFNγ, this agent still provoked the formation of complexes containing both STAT1 and STAT2 at the 24 h time point (Fig. 4). It should be emphasised that the levels of input STAT1 were variable in these experiments (because of varying levels of induction during IFN treatment), but we consider it unlikely that non-specific interactions between STAT1 and STAT2 could fully explain their co-immunoprecipitation. In accord with this, we found that ISRE-reporter activity was correspondingly increased in cells treated with IFNγ at later time points. Furthermore, analysis of the intracellular distribution of STAT1 and STAT2 following treatment with IFNs, by immunocytochemistry, revealed that both isoforms were detected in the cytosolic and nuclear compartments of EndoC-βH1 cells (Fig. 5) although the majority of STAT2 was still retained in the extra-nuclear compartment under these conditions. For comparison, we also examined the intracellular distribution of each STAT isoform in cells exposed to IFNs for only 30 min. As expected, this revealed the migration of STAT1 from the cytosolic to the nuclear compartments after exposure to each of the four IFNs. By contrast, although STAT2 also accumulated in the nucleus soon after the treatment of EndoC-βH1 cells with IFNα, λ1 or λ2, this did not occur in cells treated with IFNγ where it remained localised mainly within the cytosol (Fig. 5 and Supplementary Fig. 1). To verify the subcellular localisation of unphosphorylated STAT molecules, 3D Z-stacks were constructed from confocal images to reveal the planes above, below and within the nuclei (not presented).

### Desensitisation of STAT phosphorylation in beta-cells following interferon treatment

**Figure 6:**
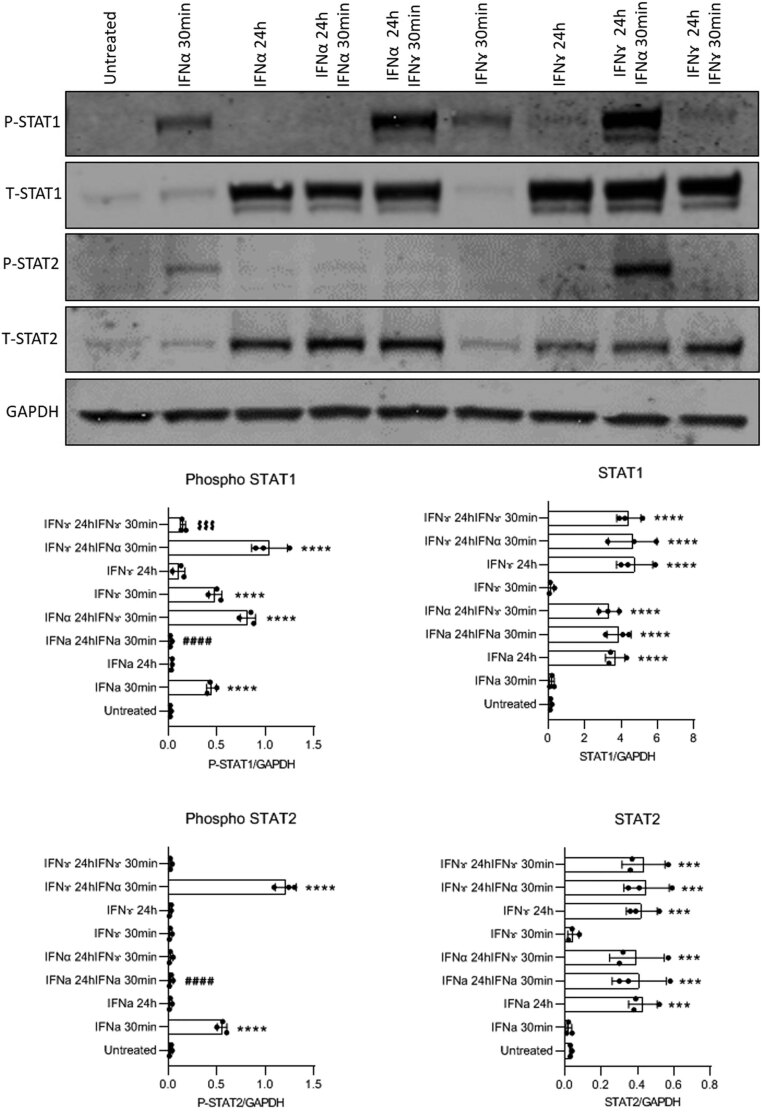
EndoC-βH1 were treated with either IFNα – 1000 U/mL or IFNγ – 20 ng/mL for 24 h. Cells were washed with PBS and retreated with either IFNα (1000 U/mL) or IFNγ (20 ng/mL) for a further of 30 min. Protein was extracted at the end of each incubation period and Western blotting performed using anti-pSTAT1, anti-total STAT1, anti-pSTAT2 and anti-total STAT2. Representative blots are presented but densitometric traces were obtained from a minimum of three separate experiments in each case (lower panels). GAPDH was used as a loading control. ^****^P  < 0.0001; ^**^P  < 0.01; ^*^P  < 0.05.

**Figure 7:**
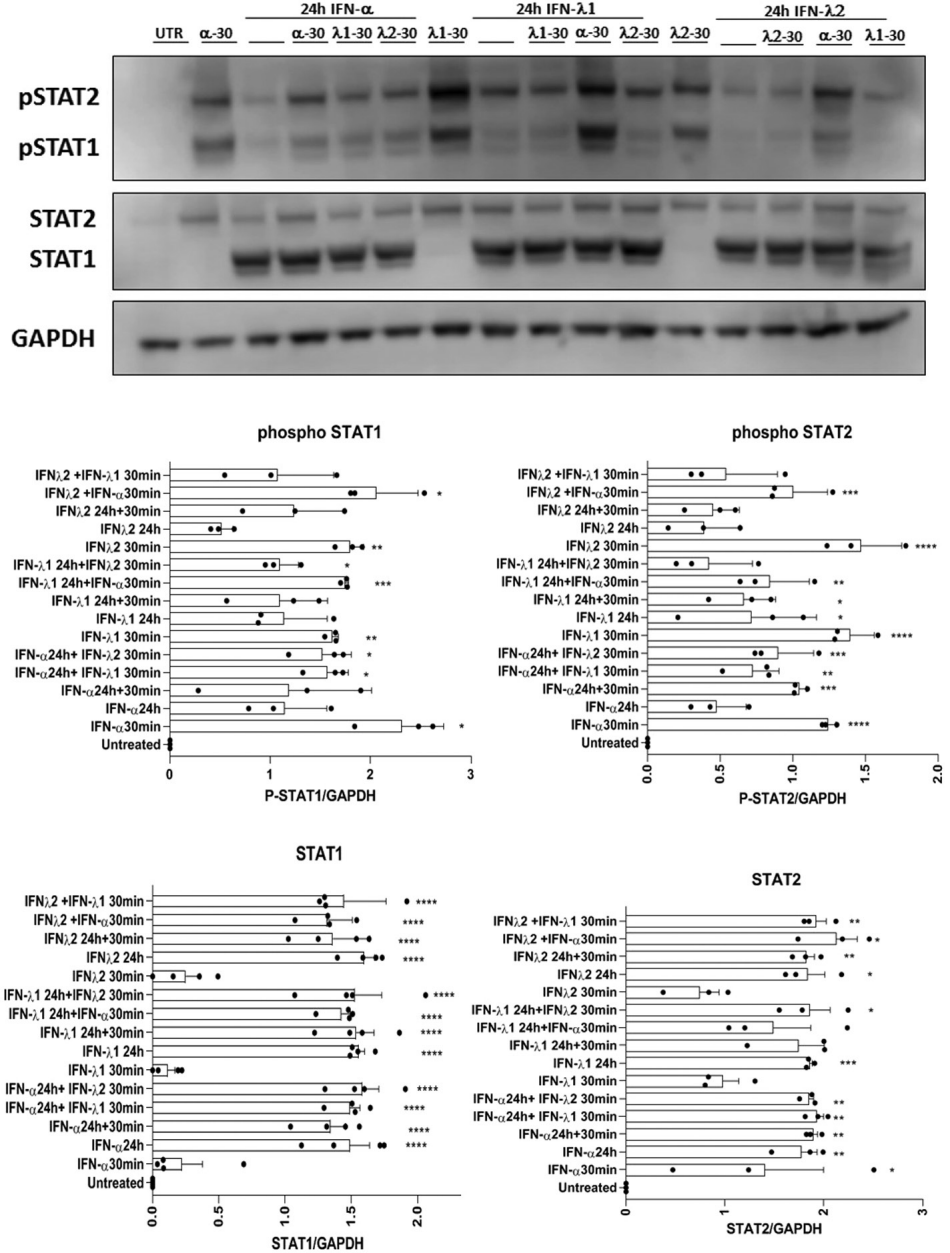
EndoC-βH1 were treated with either IFNα (1000 U/mL) or IFNλ1 or IFNλ2 (20 ng/mL) for 24 h. After this time, cells were washed and retreated with either IFNα (1000 U/mL) or IFNλ1 or IFNλ2 (20 ng/mL) for a further 30 min. Protein was extracted at the end of the incubation and Western blotting performed using anti-pSTAT1, anti-total STAT1, anti-pSTAT2 and anti-total STAT2. GAPDH was used as a loading control (representative blots are shown; *n*  = 3).

In view of the finding that treatment of EndoC-βH1 cells with IFNs leads to a large increase in the expression of STAT1 and STAT2, it was important to establish whether this is associated with an altered responsiveness to the stimuli. Accordingly, cells were exposed to IFNs for up to 24 h and then a second bolus of ligand was added and STAT phosphorylation was monitored (Figs 6 and 7). As expected, the initial addition of IFNα resulted in early STAT1/2 phosphorylation (within 30 min) which then declined but was followed by a marked upregulation of STAT1/2 expression over the subsequent 24 h period. Importantly, introduction of a further bolus of IFNα at the end of this 24 h period failed to promote any increase in either STAT1 or STAT2 phosphorylation despite the large increase in total STAT levels that had occurred (Fig. 6). Thus, it appears that a process of desensitisation follows from the initial IFN stimulus which prevents further STAT phosphorylation despite the elevated levels of protein available. To examine the specificity of this desensitisation, the cells were also challenged with a different IFN (IFNγ) at the end of the initial 24 h incubation period. When IFNγ was introduced following an earlier exposure to IFNα, a rapid and large increase in STAT1 phosphorylation occurred within 30 min, thereby revealing that the desensitisation response was not fully heterotypic (Fig. 6).

**Figure 8:**
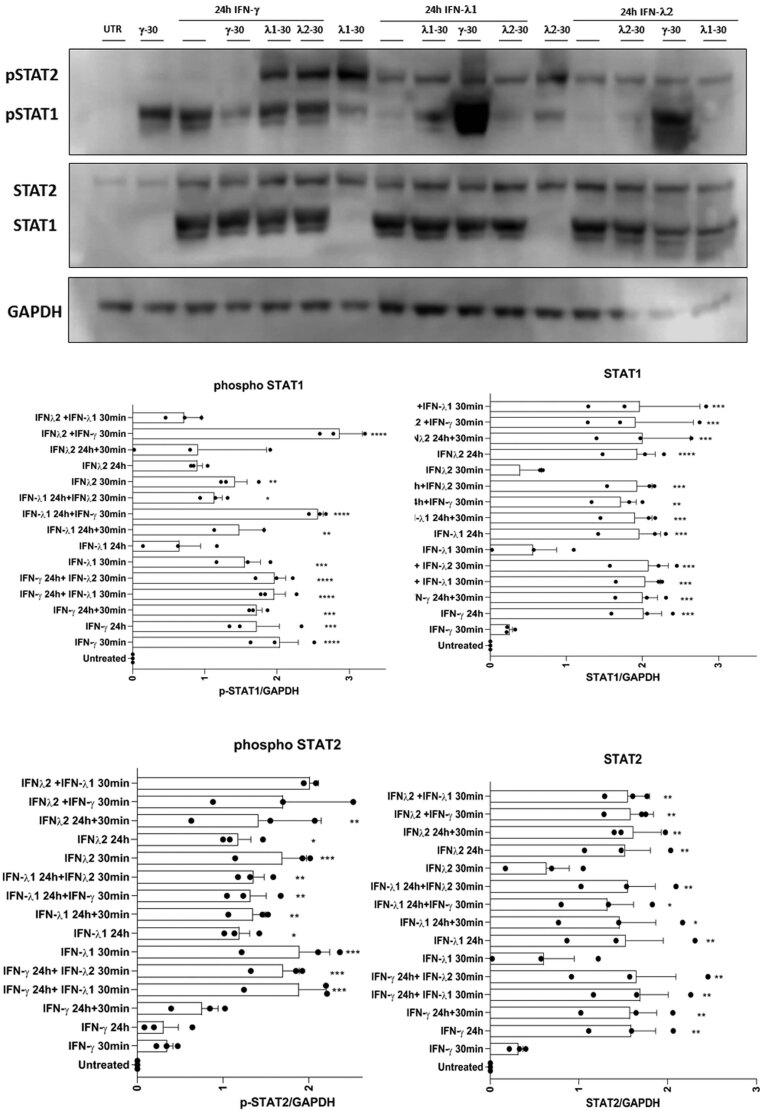
EndoC-βH1 were treated with either IFNγ (1000 U/mL) or IFNλ1 or IFNλ2 (20 ng/mL) for 24 h. After this time, cells were washed and retreated with either IFNγ (1000 U/mL) or IFNλ1 or IFNλ2 (20 ng/mL) for a further 30 min. Protein was extracted at the end of the incubation and Western blotting performed using anti-pSTAT1, anti-total STAT1, anti-pSTAT2 and anti-total STAT2. GAPDH was used as a loading control (representative blots are shown; *n*  = 3).

In order to verify these conclusions, the experiment was repeated in reverse order with the initial stimulus being IFNγ, while the second stimulus (added after a period of 24 h) was then with IFNα. An identical pattern was seen with the initial increase in STAT1 phosphorylation caused by IFNγ leading to complete loss of any further response upon subsequent re-introduction of the ligand. However, addition of IFNα following the initial IFNγ treatment led to a marked increase in STAT1/2 phosphorylation (Fig. 6). Interestingly, when a similar experiment was conducted in which IFNλ1 or IFNλ2 was employed during the initial exposure and the responses to either IFNα or IFNλ1 or 2 were then measured 24 h later, and it was found that cross-desensitisation had occurred (Fig. 7). Thus, prior exposure to IFNα caused an attenuation of STAT1/2 phosphorylation when the cells were exposed subsequently to a bolus of either IFNλ1 or IFNλ2. Such cross-desensitisation was less effective under the reverse conditions since initial exposure to either IFNλ1 or IFNλ2 was associated with, at best, only a modest reduction in the extent of STAT1/2 phosphorylation upon subsequent exposure to IFNα. IFNλ1 and IFNλ2 were able to desensitise the response to each other (Figs 7 and 8) whereas they did not lead to any desensitisation of the response to IFNγ (Fig. 8).

**Figure 9:**
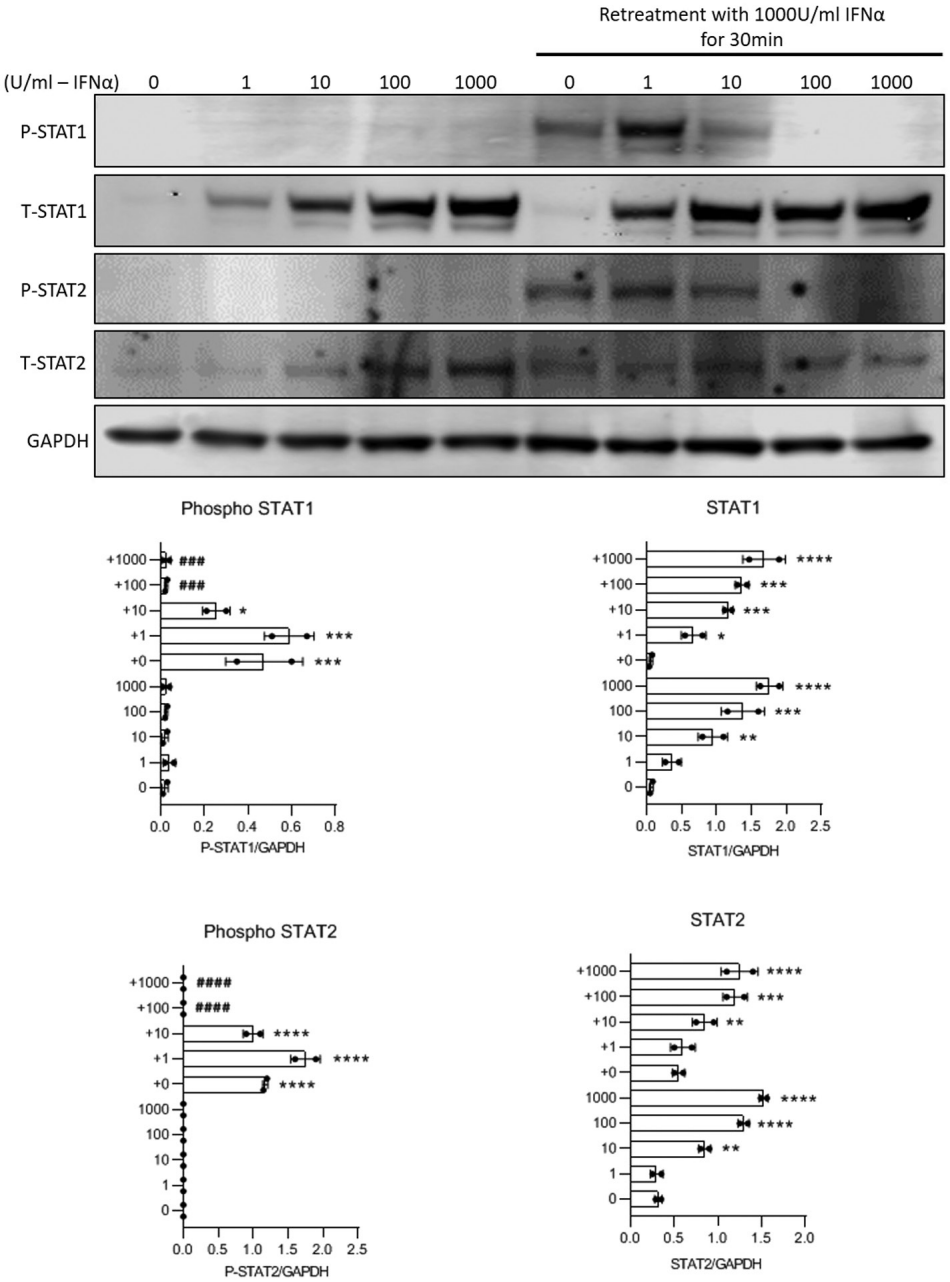
EndoC-βH1 were treated with increasing concentration of IFNα (1, 10, 100 or 1000 U/mL) for 24 h. Cells were then washed with PBS and treated with 1000 U/mL IFNα for a further of 30 min. Cells were lysed and Western blotting performed using anti-pSTAT1, anti-total STAT1, anti-pSTAT2 and anti-total STAT2. GAPDH was used as a loading control. Representative blots are presented but densitometric traces were obtained from two separate experiments in each case (lower panels). ^****^P  < 0.0001; ^***^P  < 0.001; ^**^P  < 0.01; ^*^P  < 0.05.

To establish the dose–response relationship of IFNα-induced desensitisation, cells were exposed initially to increasing concentrations of IFNα for 30 min prior to lysis and extraction (Fig. 9). Under these conditions, STAT1 and STAT2 phosphorylation were each increased over the range 1–1000 U/mL IFNα. Similarly, total STAT1 and STAT2 levels were also increased over the same concentration range when assessed after 24 h of incubation. Upon subsequent addition of the highest concentration of IFNα at the end of the initial 24 h period, the extent of STAT1 and STAT2 phosphorylation diminished in parallel with the initial IFNα concentration employed. Thus, low doses of IFNα (1–10 U/mL) caused a modest initial phosphorylation of STAT1 and STAT2 and subsequent introduction of a bolus of 1000 U/mL IFNα then resulted in a renewed phosphorylation of STAT1 and STAT2. However, no renewal of this response was observed when higher doses of IFNα (100–1000 U/mL) were employed initially (Fig. 9).

## Discussion

IFNα is secreted by virally infected cells as an early response designed to signal the presence of infection to neighbouring cells. This then allows these cells to respond by upregulating critical anti-viral response genes, to limit the rate and extent of viral spread (Murira & Lamarre 2016). It is widely accepted that such mechanisms operate in the islets of people with type 1 diabetes since there is firm evidence that both enteroviral infection and IFNα secretion occur in the islets during the development of beta-cell autoimmunity (Foulis *et al.* 1987, Jean-Baptiste *et al.* 2017, Craig *et al.* 2019, Akhbari *et al.* 2020). In addition, it is evident that other IFNs (including IFNγ and both isoforms of IFNλ) are also present within the islet milieu during the progression of type 1 diabetes and that, collectively, these (and other) cytokines determine the rate and extent of beta-cell loss (Huhn *et al.* 2008, Colli *et al.* 2020). Hence, as viral infections and insulitis develop, beta-cells are required to mount coordinated responses to a range of signals emanating from the activation of multiple IFN receptors. Surprisingly, the temporal changes associated with IFN action and the mechanisms by which signal integration is achieved have received only limited attention in β-cells although the molecular events associated with the activation of each individual signalling pathway have been studied in much greater detail.

Canonically, it is considered that type I (IFNα) and type III (IFNλ) interferons promote gene transcription by inducing the formation of STAT1/2 heterodimers, while type II interferon (IFNγ) preferentially promotes STAT1 homodimer formation (Platanias 2005, Majoros *et al.* 2017, Stanifer *et al.* 2019). Accordingly, the profile of genes induced by each class of interferons also differs and this reflects the ability of each of the fully assembled pSTAT complexes to bind differentially to relevant promoter regions within target DNA (Ramana *et al.* 2000, Michalska *et al.* 2018). These processes can be interrogated experimentally by transfection of cells with reporter constructs containing either ISRE or GAS, respectively. In the present work, we have used such promoter constructs to control the expression of luciferase enzymes following exposure of human EndoC-βH1 cells to each class of interferon. The data obtained reveal a greater degree of promiscuity in signalling than might be expected from the canonical interpretation.

First, we report that the functional outcomes measured when beta cells are exposed to individual interferons vary temporally according to the nature of the initial agonist used. This is seen most clearly when comparisons are made of the luciferase reporter activity measured after treatment of cells with type I interferons vs those exposed to type II interferon for different periods of time. In cells treated with IFNα, the ISRE promoter was activated significantly within 2 h, consistent with early increases in pSTAT1 and pSTAT2 detected under such conditions. Moreover, continued exposure of the cells for a period of 24 h resulted in a much greater increase in luciferase activity implying a sustained activation of signalling. However, this occurred despite the observation that, by 24 h, the levels of pSTAT1 and pSTAT2 had declined dramatically from their initial peak. More surprisingly, we found that IFNγ also induced a marked rise in ISRE-driven luciferase activity after 24 h, although this was not seen within the first 2 h of exposure. This late-developing enhancement of ISRE activity mediated by IFNγ occurred without any concomitant increase in pSTAT2 levels, which are normally considered critical to drive ISRE responses. Interestingly, IFNγ has also been shown to increase ISRE response in other cell types (Bluyssen *et al.* 1995, Guinn *et al.* 2017). Taken together, these findings imply that, in EndoC-βH1 cells, increases in pSTAT2 are not absolutely required to deliver enhanced transcriptional activity from ISRE-responsive promoters. As such, it seems possible that, at least in these cells, the generation of pSTAT2 is more intimately involved in regulating the time course over which ISRE-responses develop, rather than in controlling their absolute specificity.

The situation arising from activation of the GAS reporter in cells treated with interferons was markedly different. Here, much greater specificity was apparent in that neither IFNα nor IFNλ was able to induce GAS-regulated luciferase activity, either early after exposure or during more chronic treatment. By contrast, IFNγ caused a rapid activation of the GAS promoter in EndoC-βH1 cells (within 2h) which then increased markedly during more prolonged treatment. Since this response was accompanied by early and selective phosphorylation of STAT1, the data are consistent with the view that GAS activity requires the formation of a complex containing pSTAT1 homodimers. However, if this is the case, then it must also be true that such complexes do not form when cells are exposed to IFNα or IFNλs, even though phosphorylation of STAT1 can be detected readily under these conditions. This might be due to the preferential formation of pSTAT1/pSTAT2 heterodimers upon exposure to IFNα/λ (perhaps reflecting the relative abundance of each isoform and/or their respective binding affinities) or the failure to recruit a relevant additional binding partner that is required to drive transcriptional activation in concert with pSTAT1. Whatever the precise mechanism, the results reveal that the integrity of canonical downstream signalling pathways is compromised in IFNγ-treated beta-cells during chronic stimulation.

A second anomaly is revealed by study of the total STAT1/2 levels in EndoC-βH1 cells during exposure to IFN isoforms. Thus, whereas the early generation of pSTAT1 and/or pSTAT2 is determined by the precise IFNs employed, the later induction of total STAT1/2 expression is not. This is illustrated by considering the responses seen during exposure of cells to a single bolus of IFNγ. Under these conditions, the early formation of pSTAT1 was detected but phosphorylation of STAT2 did not occur. Despite this, a dramatic and sustained elevation in total STAT2 levels developed over 24 h. These results imply very strongly that transcriptional activation of the STAT2 gene is not consequential to the operation of a feed-forward mechanism that requires the initial formation of pSTAT2. Paradoxically, it also follows that formation of pSTAT1 homodimers is equally unlikely to drive the response since, as noted above, the transcriptional activation of GAS sequences (reflecting the formation of pSTAT1 homodimers) was not detected during exposure of EndoC-βH1 cells to IFNα even though total STAT2 levels were increased under these conditions. Thus, we find that IFNs regulate STAT1/2 signalling by two apparently independent pathways in human beta-cells. One of these involves a canonical mechanism associated with early phosphorylation of relevant STAT isoforms, while the second operates separately and yields a rise in the total levels of both STAT1 and STAT2.

Currently, the functional consequences of the sustained upregulation of unphosphorylated STAT isoforms are unclear but it may be significant that evidence in other cell types implies that STAT molecules can drive MHC class I expression despite persisting in a de-phosphorylated form (Poat *et al.* 2010, Srivastava *et al.* 2015). Since the sustained hyper-expression of MHC-I is a defining feature of islet cells in type 1 diabetes (Richardson *et al.* 2016, Marroqui *et al.* 2017, Wyatt *et al.* 2019) (and occurs in EndoC-βH1 cells following exposure to type I, II or III interferons) (Marroqui *et al.* 2017, Colli *et al.* 2020), it is tempting to hypothesise that this is mediated by the long-term increase in unphosphorylated STAT1/2 seen following exposure of the cells to each IFN isoform.

The third area of importance relates to our finding that during chronic stimulation of EndoC-βH1 cells, desensitisation of interferon responses occurs. This is important because, in the context of type 1 diabetes, islet cells are likely to be exposed to elevated interferon levels chronically as inflammation develops. It is also of importance because the desensitisation response displays a measure of agonist-specificity although this is incomplete. Thus, in our studies, exposure of EndoC-βH1 to IFNα caused a marked loss of response (measured as STAT phosphorylation) when the second bolus of this cytokine was introduced 24 h later. The equivalent response to IFNλs was similarly compromised (suggesting cross-desensitisation) whereas, when IFNγ was introduced following prior exposure to IFNα, the phosphorylation of STAT1 was increased above that achieved with a single exposure to IFNγ alone. Bluyssen *et al.* have also reported that IFNγ-induced ISRE activity was increased when cells were pre-exposed to IFNα and have suggested that this may reflect an increase in the formation of ISGF3 (Bluyssen *et al.* 1995).

Study of the dose–response relationship for IFNα-induced desensitisation in EndoC-βH1 cells revealed that the extent achieved correlates with the magnitude of the initial cellular response. Thus, treatment of the cells with a high concentration of IFNα (1000 U/mL) caused complete desensitisation to the subsequent addition of the cytokine (for a period of at least 24 h). By contrast, pre-exposure to a lower dose of IFNα (<100 U/mL) was only partially effective, thereby allowing a further (albeit attenuated) response when IFNα was re-introduced.

Importantly, although the early responses to IFNs (e.g. STAT phosphorylation) are attenuated (or lost completely) during desensitisation, our results also reveal that the longer-term actions of each IFN are not desensitised in β-cells. Thus, total STAT1/2 levels remain elevated for long periods following the initial period of exposure to IFNs, suggesting that the consequences of this secondary response (which, as emphasised above, may include the induction of MHC class I hyper-expression) are persistent even under conditions when the acute responses are fully desensitised. Similarly, Yao *et al*. (2017) reported that total STAT1 levels were sustained for several days after treatment of murine macrophages with IFNs.

We have not addressed fully the molecular mechanisms by which the early desensitisation to IFN treatment occurs. However, the fact that it displays only partial agonist selectivity implies that changes in receptor expression at the cell surface are unlikely to be a primary cause. Rather, it seems more probable that downstream signalling events are involved and, in other cell types, the levels of expression of a key interferon-sensitive gene, ubiquitin-specific peptidase 18 (USP18), have been implicated in controlling IFNα-mediated desensitisation (Mudla *et al.* 2020). Consistent with this, we and others have found that USP18 levels are increased dramatically in EndoC-βH1 cells in response to IFNα (Marroqui *et al.* 2017) and it will be important in future studies to explore whether this enzyme (and/or a range of additional ‘negative regulators’ such as PIAS proteins and SOCS1, which are currently under investigation in parallel studies), is responsible for controlling the desensitisation response.

Overall, the present data imply that during the progression of insulitis, the capacity of beta-cells to mount a response to IFNs will vary according to the prevailing cytokine concentration. However, this does not simply reflect the levels of the individual cytokines but it is also influenced markedly by their order of presentation. This is important because the evidence accumulated to date implies that enteroviral infection persists in selected beta-cells at a relatively low level during the progression of type 1 diabetes (Krogvold *et al.* 2015, Dunne *et al.* 2019). Accordingly, it seems likely that IFNα elaboration within the islets will be similarly low during the progression of such infections. On this basis, we propose that the balance achieved between exposure to a modest level of IFNα (i.e. at a level below that required for full receptor occupancy) and the ability to sustain a sub-maximal cellular anti-viral response despite the tendency for desensitisation may be critical to the development of chronic enteroviral infection over prolonged periods.

## Supplementary materials

Supplementary Figure 1

## Declaration of interest

The authors declare that there is no conflict of interest that could be perceived as prejudicing the impartiality of the research reported.

## Funding

The authors are grateful for financial support from Diabetes UK (project grant 15/0005156) MRC (project grant: MR/P010695/1) and JDRF (3-SRA-2017-492-A-N). This project has also received funding from the Innovative Medicines Initiative 2 Joint Undertaking under grant agreement No 115797 (INNODIA) and No 945268 (INNODIA HARVEST). This Joint Undertaking receives support from the Union’s Horizon 2020 research and innovation programme, ‘EFPIA’, ‘JDRF’ and ‘The Leona M. and Harry B. Helmsley Charitable Trust’. Additional support was provided to MAR via a European Foundation for the Study of Diabetes (EFSD)/Lilly Fellowship and a Society for Endocrinology early career grant.

## Data availability

The data that support the findings of this study are available from the corresponding authors upon reasonable request.

## Author contribution statement

All the authors have critically reviewed the intellectual content of the manuscript. N G M, M A R, S J R and S D designed the study. N G M, M A R and S D drafted the manuscript. S D, K A L, M B and P A carried out the experimentation. All authors approved the final version.
