# Supplementary material for: Temporal regulation of interferon signalling in human EndoC-βH1 cells
Source: J Mol Endocrinol. 2022 Apr 19;69(2):299–313. doi: 10.1530/JME-21-0224 (PMC9175560; doi:10.1530/JME-21-0224)
Supplement: Supplementary Figure 1 [file supplementary_figure_1.pdf]

Supplemantary – Fig 1

STAT1
Full Images

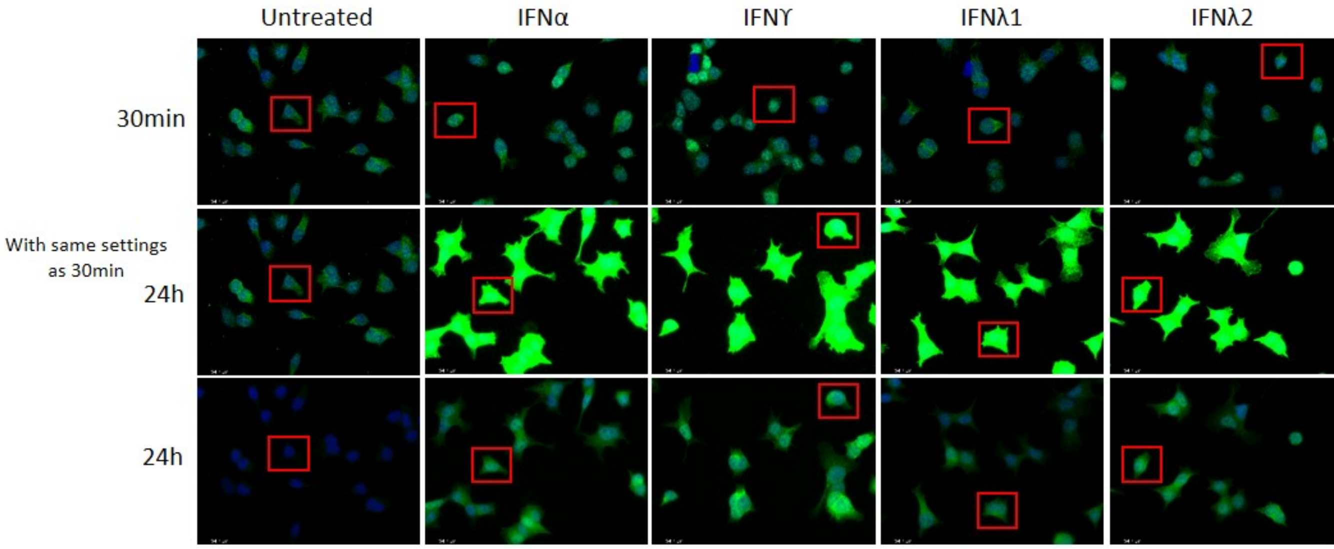

STAT2
Full Images

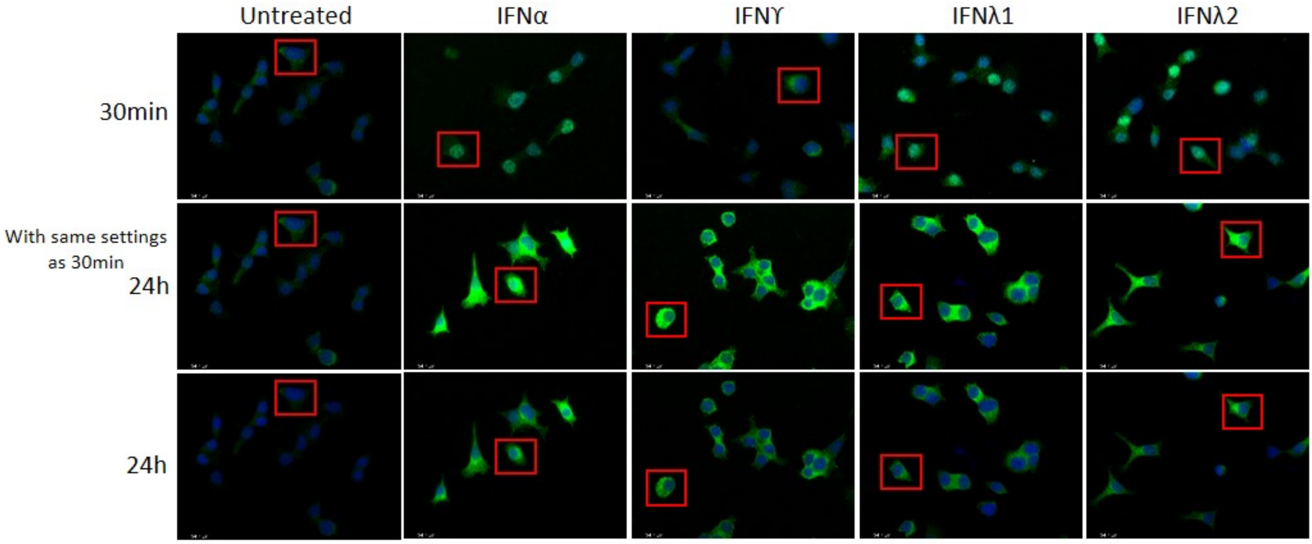

EndoC-βH1 were seeded at a density of $4X10^5$/ml and cultured for 24h. Thereafter, cells were treated with either 1000U/ml IFNα or 20ng/ml IFNɤ or 200ng/ml IFNʎ1 or IFNʎ2 for 24h. At the end of treatment, cells were fixed with 4% paraformaldehyde. Fixed cells were then permiablised and stained with anti STAT1 antibody. Images were taken using a Leica DM4000 B LED Fluorescence microscope.
The gain and exposure were same within time points but differed between 30min and 24h. Same image is used under untreated conditions to highlight the difference.
